# Potential Suitable Habitats of Chili Pepper in China under Climate Change

**DOI:** 10.3390/plants13071027

**Published:** 2024-04-04

**Authors:** Changrong Deng, Qiwen Zhong, Dengkui Shao, Yanjing Ren, Quanhui Li, Junqin Wen, Jianling Li

**Affiliations:** State Key Laboratory of Plateau Ecology and Agriculture, Academy of Agricuture and Forestry Sciences, Qinghai Key Laboratory of Vegetable Genetics and Physiology, Laboratory for Research and Utilization of Qinghai Tibet Plateau Germplasm Resources, Qinghai University, Xining 810016, China; dengchang_rong@126.com (C.D.); 13997135755@163.com (Q.Z.); 2006990015@qhu.edu.cn (D.S.); renyan0202@163.com (Y.R.); liquanhui_2008@163.com (Q.L.); 2021990055@qhu.edu.cn (J.W.)

**Keywords:** chili pepper, *Capsicum annuum* L., climate warming, MaxEnt, potential distribution

## Abstract

Chili pepper (*Capsicum annuum* L.) is extensively cultivated in China, with its production highly reliant on regional environmental conditions. Given ongoing climate change, it is imperative to assess its impact on chili pepper cultivation and identify suitable habitats for future cultivation. In this study, the MaxEnt model was optimized and utilized to predict suitable habitats for open-field chili pepper cultivation, and changes in these habitats were analyzed using ArcGIS v10.8. Our results showed that the parameter settings of the optimal model were FC = LQPTH and RM = 2.7, and the critical environmental variables influencing chili pepper distribution were annual mean temperature, isothermality, maximum temperature of the warmest month, and precipitation of the warmest quarter. Under current climate conditions, suitable habitats were distributed across all provinces in China, with moderately- and highly-suitable habitats concentrated in the east of the Qinghai–Tibetan Plateau and south of the Inner Mongolia Plateau. Under future climate scenarios, the area of suitable habitats was expected to be larger than the current ones, except for SSP126-2050s, and reached the maximum under SSP126-2090s. The overlapping suitable habitats were concentrated in the east of the Qinghai–Tibetan Plateau and south of the Inner Mongolia Plateau under various climate scenarios. In the 2050s, the centroids of suitable habitats were predicted to shift towards the southwest, except for SSP126, whereas this trend was reversed in the 2090s. Our results suggest that climate warming is conductive to the cultivation of chili pepper, and provide scientific guidance for the introduction and cultivation of chili pepper in the face of climate warming.

## 1. Introduction

Vegetables are rich in essential nutrients, such as antioxidants, vitamins, minerals, and dietary fiber, and are crucial for maintaining overall well-being [1]. Incorporating a diverse range of vegetables into our daily meals has been proven to bolster our immune system, reduce the risk of chronic ailments, and contribute to a healthier and more prolonged lifespan [2]. This recognition has led to a significant increase in demand for vegetables, with consumption rates doubling over the past two decades, rising from 0.55 billion tons in 1997 to 1.09 billion tons in 2017 [3].

However, as the frequency and intensity of extreme weather have increased and are expected to accelerate with further climate warming, there is a growing concern for the adverse and possibly irreversible impacts on Earth’s organisms and ecosystems [4]. Agricultural systems, including vegetable production, are highly dependent on specific environmental conditions, making them particularly vulnerable to climate change [5,6,7]. Estimates indicated that there could be an average reduction of 17% in crop yields for each one-degree Celsius rise in temperature [8]. Climate change affects the growth and production of crops by altering the distributional suitability of crops. Therefore, climate warming not only affects farmers’ incomes, but also poses a threat to global food security [9].

Vegetables mostly require a mild temperature for their growth and development, and are highly sensitive to climate fluctuations [10]. Climate vagaries, such as heatwaves, droughts, and floods, directly affect vegetable production at any stage of the crop growth cycle, from initial growth to pollination, flowering, fruit setting, and yield development. However, previous studies have primarily focused on the effects of climate change on the distribution of staple crops, such as wheat [11], rice [12], maize [13], and soybean [14], with less attention to vegetable crops [15]. Given the crucial role of vegetable crops in the global food system, there is a pressing need to identify how climate change influences their distribution. This work will provide valuable insights for scientific cultivation practices, effective assessments of agricultural disaster risks, and sustainable agricultural strategies to ensure food security in the face of changing climatic conditions [16,17].

Species distribution models (SDMs) are powerful tools for simulating the geographic distribution of species based on the available distributional information and corresponding environmental data, and have been widely applied in the prevention of invasive species [18], and the cultivation [19] and protection [20] of species. Among the available SDMs, the maximum entropy model (MaxEnt) outperforms the others for its high accuracy and stability, rapid calculation, and flexible operation [21,22].

Chili pepper (*Capsicum annuum* L.), one of the oldest domesticated cash crops, originated in Central and South America and has been cultivated extensively all over the world, with the current cultivation area reaching approximately 3.8 million hectares [23,24]. South Asia is globally recognized as the most prominent region for chili pepper cultivation, accounting for approximately 55% of the total world production. India contributes the next largest proportion (38%), followed by China (7%), while Pakistan, Peru, and Bangladesh collectively contribute 5% [24].

Chili pepper was introduced to China at the end of the 16th century [25]. After over 400 years of cultivation and culinary development, it has become an integral vegetable and spice of local cuisine in China for its nutritional values and diverse flavors [26]. Due to its short growth cycle, low production cost, and high market demand [26], many regions in China have introduced chili peppers for open-field cultivation to increase revenue. Nowadays, China is the largest fresh chili pepper producer with an annual production exceeding 18 million tons [24]. Chili pepper is playing a crucial role in the revitalization of rural areas. Nonetheless, chili pepper is a thermophilic vegetable [27], and reckless introduction to unsuitable regions may lead to a series of issues, such as increased production costs, decreased yields and lower profitability [28]. Additionally, the warming climate has been resulting in more frequent extreme weather, which compromises the growth of chili pepper and dramatically decreases yields [29]. Therefore, it is imperative to determine the suitable habitats for chili pepper under climate change, in order to provide scientific guidance for cultivation practices and ensure sustainable development of chili pepper industry.

In this study, the MaxEnt model was optimized and utilized to predict the current and future suitable habitats of chili peppers for open-field cultivation under different climate scenarios, as well as to identify the predominant environmental variables influencing chili pepper distribution. Subsequently, spatiotemporal changes and centroid shifts in the suitable habitats were analyzed using ArcGIS. This study will provide a theoretical basis for the introduction and cultivation of chili peppers.

## 2. Results

### 2.1. Screening of Distribution Points and Environmental Variables, and Accuracy of MaxEnt Prediction

**Figure 1:**
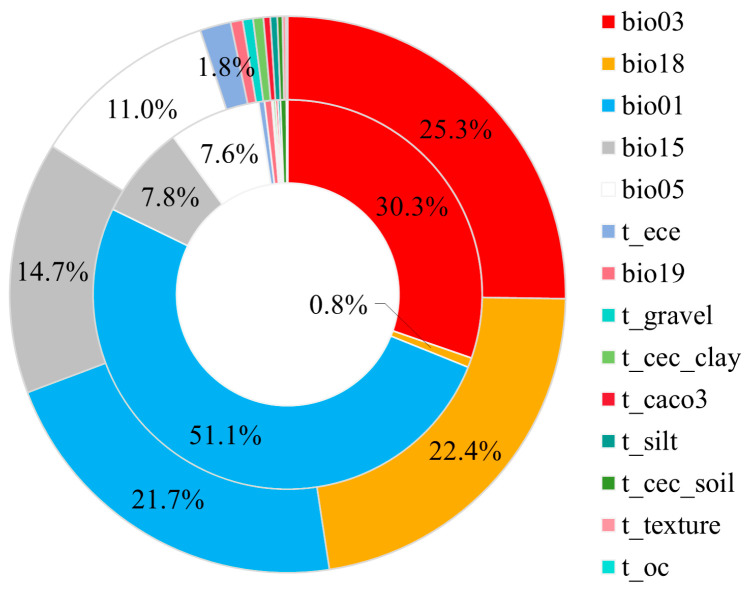
Percent contribution (outer ring) and permutation importance (inner ring) of environmental variables.

After screening, 369 of 732 distribution points and 14 (six climate and eight topsoil variables) of 36 environmental variables were selected for MaxEnt prediction (Figure 1). Based on the results output by Kuenm, when FC and RM were separately set to LQPTH and 2.7, we found ΔAICc was 0, which was the best candidate mode for MaxEnt.

The current suitable habitats of chili pepper were simulated using the optimal model based on the screening distribution points and environmental variables. The simulation results showed that the training omission rate was very close to the predicted omission (Figure S1), and the average training AUC and TSS were 0.974 and 0.927, indicating that the reconstructed model was highly reliable and qualified for the following predictions.

### 2.2. Critical Environmental Variables Affecting Chili Pepper Distribution

The critical environmental variables affecting the distribution of chili pepper were determined via the MaxEnt jackknife test. As shown in Figure 1, the cumulative contributions and permutation importance of climate variables were 95.8% and 98.2%, indicating that the distribution of chili pepper was primarily affected by climate rather than soil. The results of the jackknife test of variable importance showed that bio01 (annual mean temperature), bio05 (maximum temperature of the warmest month), bio03 (isothermality), and bio18 (precipitation of the warmest quarter) had higher weights in single variables analysis (Figure S2), with a cumulative contribution rate of 80.4% and permutation importance of 89.8% (Figure 1), indicating that these variables possessed more effective information in chili pepper distribution than the others. Among the four variables, bio01 was the environmental variable with highest gain when used in isolation, which appeared to have the most useful information by itself; bio03 was the environmental variable that decreased the gain the most when it was omitted, which appeared to have the most information that was not present in the others (Figure S2).

The relationships between the distributional probability and environmental variables were identified using single-factor response curves output by the MaxEnt model. The suitable ranges [distribution probability > MTSPS (0.1575)] of bio01, bio03, bio05, and bio18 for chili pepper were 4.08–24.75 °C, 21.61–48.90%, 23.75–33.62 °C, and >224.85 mm, respectively (Figure S3).

### 2.3. Current Distribution of Chili Pepper

**Figure 2:**
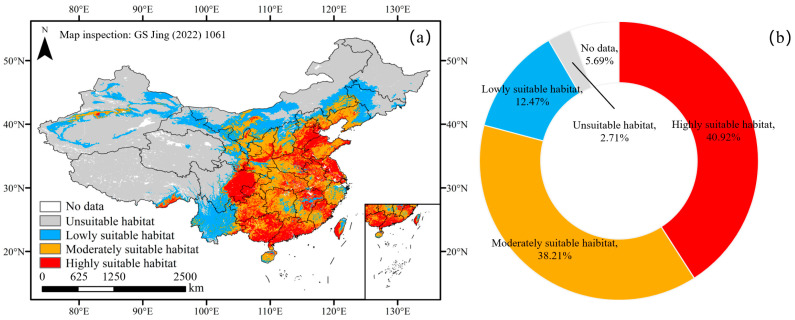
Current suitable habitats (**a**) and proportion of distribution points in different habitats (**b**) of chili pepper.

Under current climate conditions, the simulation results were highly consistent with the actual cultivation of chili pepper (Figure 2a). A total of 91.60% of the screening distribution points were located in suitable habitats, of which 79.13% were concentrated in moderately- and highly-suitable habitats (Figure 2b), indicating that the results were accurate and reliable.

**Figure 3:**
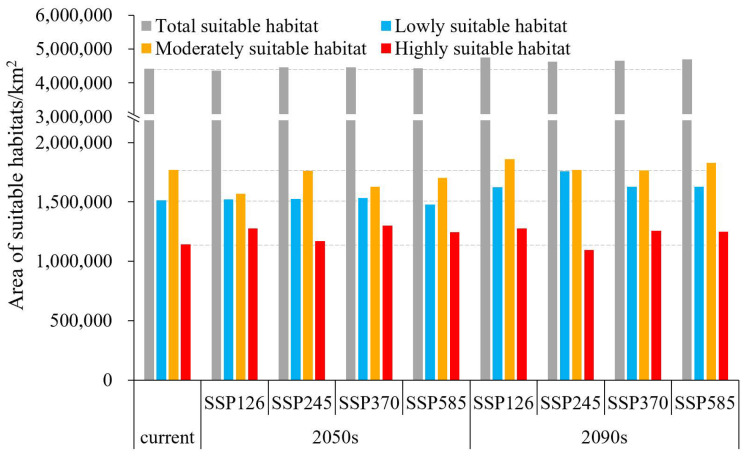
Current and future suitable area for chili pepper cultivation under different climate scenarios.

The current suitable area for chili pepper cultivation was 4,426,594.63 km^2^ in China (Figure 3), accounting for 46.11% of China’s land area and distributed across all provinces, mainly in 18° N–46° N and 108° E–126° E (Figure 2a). The moderately- and highly-suitable area was 2,913,626.88 km^2^, making up 65.82% of the total suitable area, which was concentrated in the east of the Qinghai–Tibetan Plateau and south of the Inner Mongolia Plateau. Although there were some suitable habitats for chili pepper cultivation in the Tibetan Plateau, they were mainly located in the north, east and southeast marginal regions. Among all provinces of China, Qinghai had the smallest suitable area, with only 8800.04 km^2^, and Heilongjiang was the only one without moderately- and highly-suitable habitats (Figure 2a).

### 2.4. Future Distribution of Chili Pepper

**Figure 4:**
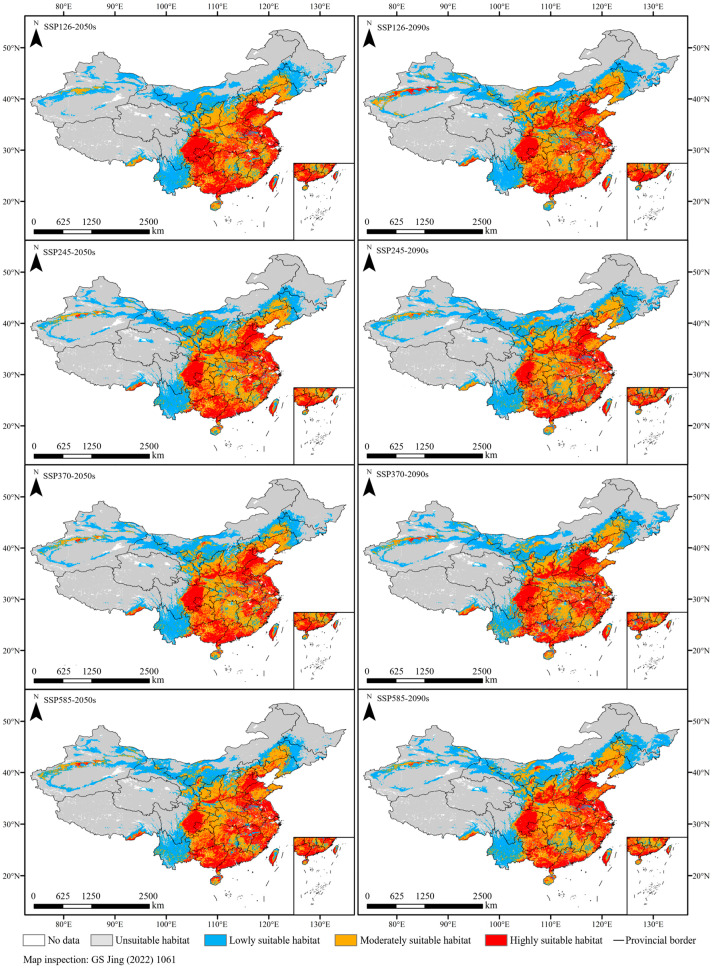
Future suitable habitats under different climate scenarios.

The potential distribution of chili pepper in the 2050s and 2090s under four climate scenarios (SSP126, SSP245, SSP370, and SSP585) were predicted using the optimal MaxEnt model (Figure 4). Under future climate scenarios, the suitable habitats of chili pepper were distributed across all provinces of China, and the moderately- and highly-suitable habitats were concentrated in the east of the Qinghai–Tibetan Plateau and the south of the Inner Mongolia Plateau, which were basically consistent with the current ones (Figure 4).

In the 2050s, the area of suitable habitats initially experienced an increase and subsequently declined in response to greenhouse gas emissions, reaching its maximum (4,462,849.42 km^2^) under SSP370 with a growth rate of 0.82%. Moreover, the moderately- and highly-suitable area peaked (2,949,743 km^2^) with a 1.24% increase under the SSP585 scenario (Figure 3). Notably, both the total suitable and the moderately- and highly-suitable area were all larger than the current levels, except for SSP126.

In the 2090s, the changes in suitable area were contrary to those observed in the 2050s, with an initial decrease followed by an increase. The area of suitable habitats expanded in different degrees compared with that of the current climate, with the highest growth rate of 7.49% under SSP126, followed by SSP585 (6.28%), SSP370 (5.08%), and SSP245 (4.43%) (Figure 3). The moderately- and highly-suitable habitats shrank by 1.68% under SSP245, and the other scenarios expanded by 3.73–7.63%.

### 2.5. Future Spatiotemporal and Centroid Changes in Suitable Habitats

**Figure 5:**
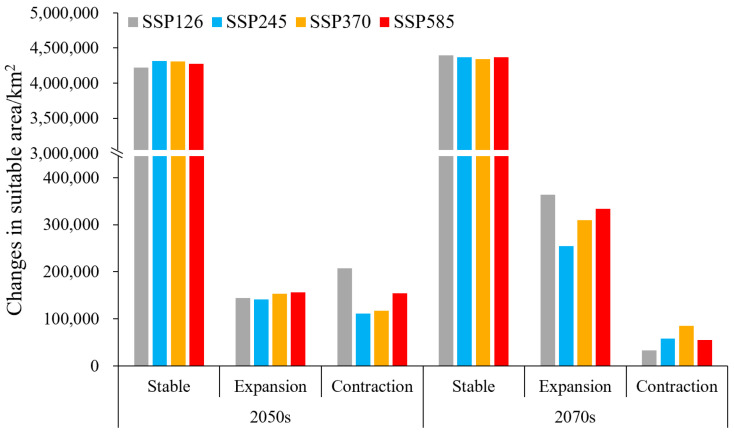
Spatiotemporal changes in the future suitable area compared with the current.

**Figure 6:**
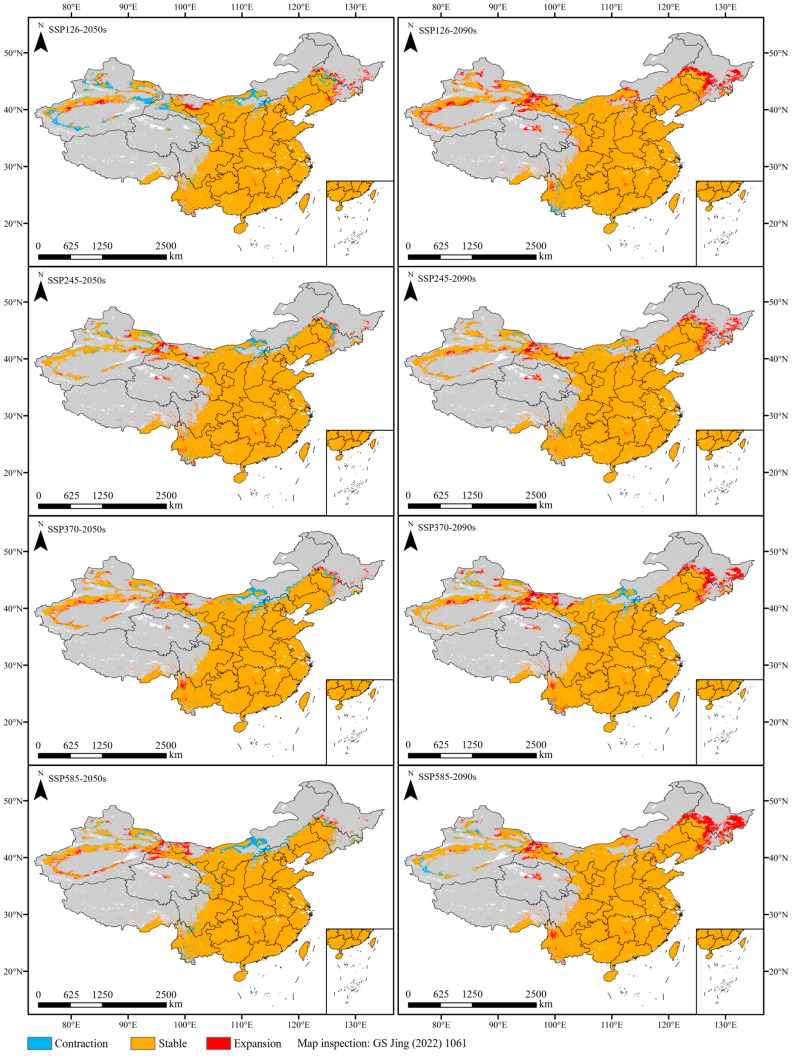
Spatiotemporal changes in the future suitable habitats of chili pepper compared with the current.

Compared with the current situation, 92.91–96.82% of suitable habitats under future climatic scenarios remained unchanged (Figure 5); they are mainly located in the south of the Inner Mongolia Plateau and the east of the Tibetan Plateau (Figure 6). The expansion area under different climate scenarios of the 2050s and 2090s was all larger than the contraction, with a 0.01–10.21-fold increase over the contraction, except for SSP126-2050s (expansion 143,826.25 km^2^, contraction 207,691.39 km^2^). Furthermore, the expansion area under future 2090s climate scenarios exceeded those of the future 2050s, reaching a maximum of 364,035 km^2^ under SSP126-2090s, whereas the contraction area was surpassed by the latter.

In the future 2050s, the expansion of suitable habitats was distributed in the west of the Inner Mongolia Plateau, and the contraction was primarily distributed in the central regions of the Inner Mongolia Plateau (Figure 6). In the 2090s, the expansion regions were concentrated in the northeast of the current ranges and the west of the Inner Mongolia Plateau, while the contraction regions were limited and uncertain. They were mainly situated in the southern Yunan Province under SSP126-2090s, and migrated to the central regions of the Inner Mongolia Plateau under the other climate scenarios (Figure 6).

**Figure 7:**
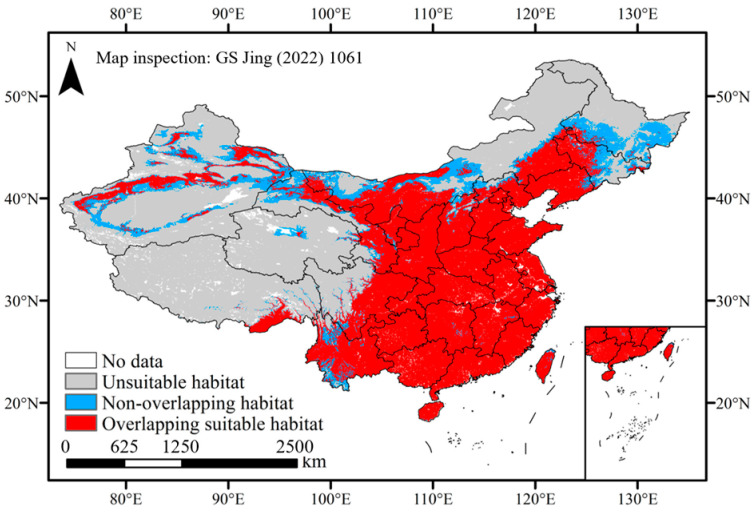
Overlapping and non-overlapping suitable habitats under climate warming.

Climate warming was anticipated to induce minor fluctuations in the distribution of pepper cultivation, with most regions likely experiencing negligible changes (Figure 6 and Figure 7). The overlapping suitable habitats, encompassing an area of 4,101,218 km², were distributed across all provinces of China and concentrated in the east of the Qinghai–Tibetan Plateau and south of the Inner Mongolia Plateau. These unchanged habitats accounted for 42.72% of China’s land area and 92.65% of the current suitable habitats, which were always suitable for chili pepper cultivation under climate change. The non-overlapping suitable habitats covered an area of 882,200 km^2^, accounting for 21.51% of the overlapping area, which was mainly distributed in the north of the overlapping ranges (Figure 7). The suitability of these regions exhibited instability in the face of climate change.

### 2.6. Centroid Shifts in Suitable Habitats

**Figure 8:**
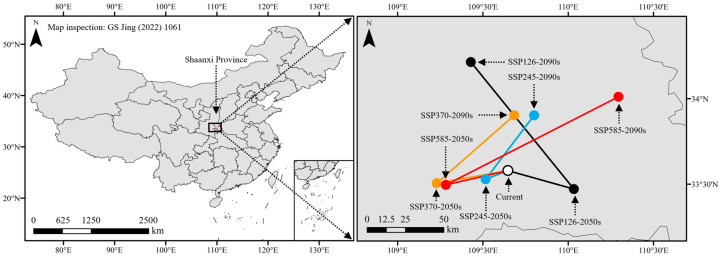
Changes in the centroids of suitable habitats under different climate scenarios.

Under the current and future (2050s and 2090s) climate scenarios, the centroids of the suitable habitats for chili pepper cultivation were all located in southeast Shaanxi Province, and the distances between the future and the current centroids (33.58° N, 109.65° E) were 13.30–76.84 km (Figure 8). In the 2050s, the centroids of suitable habitats mainly shifted to the southwest of the current ones, except SSP126, which shifted to the southeast (33.47° N, 110.03° E). In the 2090s, the changes in centroids exhibited an inverse pattern compared to those observed in the 2050s, which shifted towards the northeast of the 2050s ones, except SSP126. The centroid (34.215° N, 109.43° E) of SSP126-2090s migrated to the northwest of that in the 2050s.

## 3. Discussion

### 3.1. Dominant Environmental Variables Affecting the Distribution of Chili Pepper

For plants, temperature and precipitation have pivotal influences on their development and distribution [30]. We found that the contribution (95.8%) and the permutation (98.2%) of climate variables significantly outweighed than those of soil variables, indicating that climate had a much greater impact on chili pepper cultivation. This characteristic was reported in many plants, including cash [19] and cereal [31] crops.

In this study, we found that annual mean temperature (bio01), isothermality (bio03), maximum temperature of the warmest month (bio05) and precipitation of the warmest quarter (bio18) were the dominant variables influencing chili pepper distribution. The suitable ranges of bio01 and bio05 were 4.08–24.75 °C and 23.75–33.62 °C, respectively, indicating that the distribution of chili peppers is primarily limited to regions with warm climates, which were connected with the habit of chili peppers. Chili peppers are originally from tropical regions and require relatively high temperature for development [32]. The optimal temperature for growth is between 25–30 °C, and when the temperature is below 15 °C and above 35 °C, chili pepper growth is retarded and their yield decreases [33]. Low temperature usually induces deformed and seedless fruit [34], and high temperature inhibits fruit set [35]. Isothermality reflects the magnitude of day to night temperature oscillation relative to seasonal variation, serving as an indicator for the temperature fluctuations within months to years [36]. We found that the suitable range of bio03 was 21.61–48.9%, with an optimal value of 28.43%.

As an annual thermophilic plant, the precipitation of the warmest quarter is an important variable in determining chili pepper distribution. Our study revealed that the minimum precipitation of the warmest quarter suitable for chili pepper distribution under current climate conditions was 224.85 mm, which was basically consistent with the water requirements throughout the entire growth period of the vegetable. Previous studies have demonstrated that the total water requirement for the growth of pepper is approximately 280 mm [37,38]. The discrepancy between the minimum precipitation of the warmest quarter and the actual water requirements can be compensated through precipitation of other quarters or irrigation [39]. Moreover, we found the minimum precipitation of the warmest quarter for chili pepper increased in the future 2050s and 2090s. The reason for this is that the rise in temperature results in an elevation of evaporation, consequently leading to an augmentation in the water requirement of chili pepper [40].

### 3.2. Habitat Distribution under Climate Change

Under current climate conditions, the MaxEnt simulation results showed that the suitable habitats of chili peppers were distributed across all provinces, the predominant distribution ranged between 18° N–46° N and 108° E–126° E, and the moderately- and highly-suitable habitats were concentrated in the east of the Qinghai–Tibetan Plateau and south of the Inner Mongolia Plateau of China. The simulated suitable habitats were highly consistent with the actual cultivation of chili peppers [26,41]. For example, our results indicated that Xining prefecture-level city in Qinghai Province was unsuitable for chili pepper cultivation, while the Xunhua county of Haidong prefecture-level city bordering Xining was lowly suitable. These findings aligned with the current practices of chili pepper cultivation in these regions. In Xining, greenhouse cultivation is imperative for successful chili pepper production [41], while open-air cultivation is widely employed in Xunhua, establishing it as a crucial chili pepper production region in Qinghai [42]. Meanwhile, despite the cultivation area of chili pepper in Yunnan exceeding 1700 km^2^ and ranking among the top three in China [41], our prediction results showed that most regions of this province exhibited low suitability. Previous results have demonstrated that the production cost in Yunnan was 1.5 times higher than its neighboring Province, Guizhou, which was moderately- and highly-suitable for the vegetable cultivation, while the output in Yunnan only accounted for only 81.79% of that in Guizhou [43].

Under 2050s climate scenarios, the area of suitable habitats remained essentially unchanged compared with the current ones. However, under 2090s climate scenarios, the suitable area was all higher than the current area and reached the maximum under SSP126. Our findings indicated that the impacts of climate warming under different greenhouse gas emission modes on chili pepper cultivation varied and would become more favorable over time. This phenomenon is attributed to the thermophilic habit of chili peppers [27], which makes them sensitive to temperature, and their expansion is facilitated by the warming climate. Moreover, climate warming also leads to an increase in precipitation [44], which creates more favorable conditions for the growth of chili pepper. We found that the expansion-suitable regions were mainly distributed in the northeast and northwest of the current ranges. This phenomenon of thermophilic plants expanding towards higher latitudes in response to climate warming has been widely documented, with examples including *Lycium barbarum* L. [45], *Litsea cubeba* (Lour.) Pers [46], and *Agastache rugosa* (Fisch. & C. A. Mey.) Kuntze [20]. Although there will be more suitable habitats for chili pepper growth, this does not guarantee higher yields. Previous study has demonstrated that extreme climate warming scenarios retarded fruit morphological features and production of hot chili pepper (*C. annuum*) [47].

Climate exerts a predominant influence on the physiology, distribution, and phenology of plants, thereby potentially inducing shifts in suitable habitats [20,48]. We found that the overlapping suitable habitats of chili pepper were primary distributed in the east of the Qinghai–Tibetan Plateau and south of the Inner Mongolia Plateau under various climate scenarios. These regions consistently maintained their suitability despite climate change. Therefore, we suggest that cultivating open-air chili peppers in these regions could mitigate the impacts of climate change, facilitate vegetable growth and development, and ensure sustainable production.

### 3.3. Limitations and Prospects

In this study, MaxEnt and ArcGIS were employed to predict the suitable habitats for open-field chili pepper cultivation and analyze its changes under climate change. Our study provided scientific guidance for the introduction and cultivation of chili peppers. However, there are still some uncertainties in our study. First, plant distribution is not only influenced by climate and soil, but also by other variables, such as pests and diseases, and agronomic management [12]. Second, while environment affects plant distribution, plants are constantly adapting to the environment [49]. Plant physiological responses, including growth responses to elevated atmospheric CO_2_ and alterations in water use efficiency, are expected to mitigate the response of some plant functional types to climate change [48]. However, plant adaption is considered unchanged when SDMs predict species’ distribution. Third, the study did not consider the environmental adaptability of different chili pepper varieties, which may vary in suitable cultivation regions [50]. Therefore, the predicted potentially suitable habitats maybe deviate from the actuality. Future research should take into account these uncertainties to achieve a more accurate prediction for chili pepper cultivation under changing climatic conditions.

## 4. Materials and Methods

### 4.1. Acquisition and Processing of Chili Pepper Distribution Points and Environmental Data

**Figure 9:**
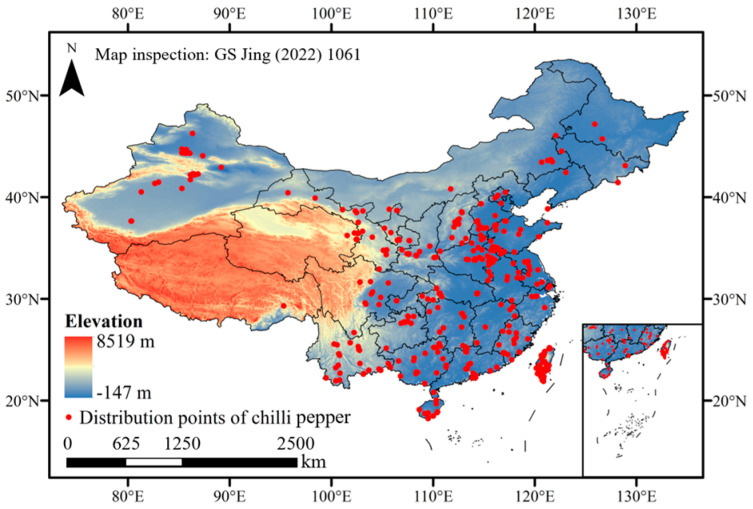
Distribution points of chili pepper in China.

The distribution points of chili pepper (*C. annuum*) in China were collected from the Global Biodiversity Information Facility (https://www.gbif.org/, assessed on 20 January 2024, Plant Science Data Center (https://www.plantplus.cn/cn/, assessed on 20 January 2024), and our survey data in 2021–2023. Overall, we collected 732 distribution points across all provinces in China (Figure 9). Because the environmental characters of greenhouse-grown chili peppers are mainly manipulated by human intervention, they are less affected by climate change, and these points were removed based on our investigations. Meanwhile, the residual points were filtered using the ‘Trim duplicate occurrence’ function of ENMtools v1.3 (http://enmtools.blogspot.com/, accessed on 13 November 2012) to avoid the overfitting of the MaxEnt model predicted results.

The current (averages for 1970–2000), future 2050s (averages for 2041–2060), and 2090s (averages for 2081–2100) climate data were downloaded from the Worldclim Database (WorldClim v2.1, https://www.worldclim.org/, assessed on 16 January 2024) with a spatial resolution of 2.5 arc-minutes (~5 km) and converted to ASCII format using ArcGIS v10.8 (https://www.esri.com/zh-cn/arcgis/, assessed on 25 April 2023). Future climatic data were determined based on the Beijing Climate Center Climate System Model (BCC-CSM) from the sixth phase of the Coupled Model Intercomparison Project (CMIP6), which included four climate scenarios based on the Shared Socio-economic Pathways (SSP126, SSP245, SSP370 and SSP585) [51]. These scenarios represented future climate scenarios with low to high greenhouse gas emissions. Each scenario had 19 climatic variables (bio01–bio19).

The topsoil data were obtained from the World Soil Database (Harmonized World Soil Database v1.2, http://www.fao.org/soilsportal/, assessed on 16 January 2024) and converted to ASCII format using ArcGIS, which contained 17 soil variables. Due to lack of future soil data, the future soil layers were considered to be consistent with the current over such a short time frame in this study [45].

There were 36 environmental variables (19 climate and 17 soil variables) initially used to construct the MaxEnt model. To avoid overfitting of the MaxEnt model causing by multicollinearity among environmental variables, the current environmental data of the screening distribution points were extracted using ArcGIS, and their correlations were examined using Pearson’s correlation analysis of SPSS (v26, https://www.ibm.com/cn-zh/spss/, assessed on 26 April 2023). The contribution rates of the current environmental variables were calculated using the jackknife analysis of MaxEnt. The variables with zero contribution were removed, and only the variables with the highest contribution were retained when the absolute correlation coefficient among them was greater than 0.7 [12].

### 4.2. Optimization and Evaluation of MaxEnt Model

Feature classes (FCs) and regularization multiplier (RM) are critical parameters affecting the accuracy of the MaxEnt (v3.4.4, https://github.com/mrmaxent/Maxent/, assessed on 20 April 2023) model, and default settings may result in model over-fitting [52]. Thus, the Kuenm package (https://github.com/marlonecobos/kuenm/, assessed on 14 December 2020) in R (v3.6.3, https://www.r-project.org/, assessed on 11 December 2020) was employed to calibrate the two parameters to select the best combination for MaxEnt [48]. 

MaxEnt contains five different FCs: linear (L), quadratic (Q), hinge (H), product (P), and threshold (T), and there is a total of 31 FCs combinations. Forty RMs (0.1–4.0 at an interval of 0.1) and 31 FCs combinations were used to generated 1240 candidate modes, and these modes were evaluated using Kuenm based on the screening environmental variables. The best candidate mode for MaxEnt was selected according to the following criteria: significant models with omission rates ≤5%, and the lowest delta-corrected Akaike information criterion (ΔAICc) values of ≤2% [53].

The other parameters of MaxEnt were selected as follows: ‘Create responsive curves’, ‘Do jackknife to measure variable importance’, ‘Out format logistic’, ‘Random seed’, ‘Random test percentage 25’, ‘Replicates 10’, ‘Replicated run type bootstrap’, ‘Write plot data’, and ‘Write background predictions’ [46]. The rest of the parameters was set to default.

The performance of the optimal MaxEnt prediction was assessed using the area under the receiver operating characteristic curve (AUC), and true skill statistic (TSS) [54] under current climate conditions. AUC and TSS values range from 0 to 1 and −1 to 1, respectively. The closer the two values are to 1, the better the model performs. AUC > 0.9 and TSS > 0.8 indicate the prediction of MaxEnt is highly reliable and excellent [55].

### 4.3. Reclassification and Calculation of Suitable Habitats

The prediction results (ASCII files) output by the optimal MaxEnt were reclassified and visualized using ArcGIS. The average logistic threshold value of maximum training sensitivity plus specificity (MTSPS) output by MaxEnt based on the current environmental variables was used to classify these results into suitability and unsuitability for chili pepper [45]. According to the suitable probability, the regions for chili pepper cultivation were divided into unsuitable habitat (0–MTSPS), lowly suitable habitat (MTSPS–0.4), moderately suitable habitat (0.4–0.6), and highly suitable habitat (0.6–1) using the reclassification function of ArcGIS. The proportion of each habitat to China overall was calculated based on its grid number, and the area of each habitat was calculated according to China’s land area [45].

### 4.4. Spatiotemporal and Centroid Changes in Suitable Habitats

SDMtoolbox v2.0 (http://www.sdmtoolbox.org/, assessed on 20 April 2023) was employed to convert the prediction ASCII files to binary files (0 unsuitability, 1 suitability) using the MTSPS threshold, and then applied to analyze the spatiotemporal changes and centroid shifts in the suitable habitats under different climate scenarios. The overlapped suitable habitats under current and future climate scenarios were determine using the ‘plus’ function of Spatial Analyst Tools. The changes in the area were calculated using the method mentioned in Section 4.3.

## 5. Conclusions

In this study, we optimized the MaxEnt model and employed it to predict the suitable habitats for open-field chili pepper cultivation in China under different climate scenarios. Our findings showed that annual mean temperature, isothermality, maximum temperature of the warmest month, and precipitation of the warmest quarter were crucial environmental variables influencing chili pepper distribution. Under current and future climate scenarios, suitable habitats were distributed across all provinces in China, with the moderately- and highly-suitable habitats concentrated in the east of the Qinghai–Tibetan Plateau and south of the Inner Mongolia Plateau. Notably, the areas of suitable habitats under future climate scenarios were all larger than the current ones, except for SSP126-2050s. The expansion habitats were mainly distributed in the west of the Inner Mongolia Plateau and the northeast of the current ranges. Moreover, the overlapping suitable habitats with stable suitability were primarily distributed in the east of the Qinghai–Tibetan Plateau and south of the Inner Mongolia Plateau under various climate scenarios. The centroids of suitable habitats shifted to the southwest in the 2050s, except for SSP126, whereas this trend was reversed in the 2090s. Our results provide guidance for chili pepper growers in selecting suitable cultivation regions while mitigating the adverse impacts of climate change. In order to attain more stable yields, we suggest selecting and cultivating varieties that possess adaptability to environmental fluctuations.

## Data Availability

Data is contained within the article.

## Supplementary Materials

The following supporting information can be downloaded at https://www.mdpi.com/article/10.3390/plants13071027/s1, Figure S1: Average omission and predicted area for chili pepper; Figure S2: Jackknife of regularized training gain for chili pepper; Figure S3: Response curves of the critical environmental variables.
