# Supplementary material for: Potential Suitable Habitats of Chili Pepper in China under Climate Change
Source: Plants (Basel). 2024 Apr 4;13(7):1027. doi: 10.3390/plants13071027 (PMC11013778; doi:10.3390/plants13071027)
Supplement: Supplementary file 1 [file plants-13-01027-s001.zip › plants-2920905-supplementary.pdf]

Supplementary Materials

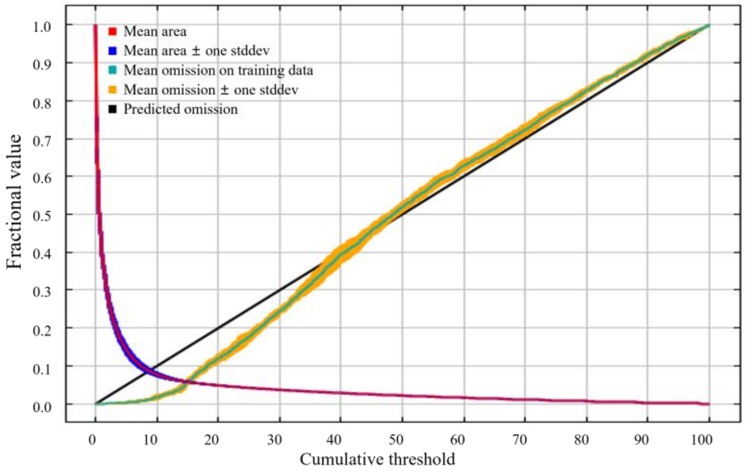

Figure S1. Average omission and predicted area for chili pepper.

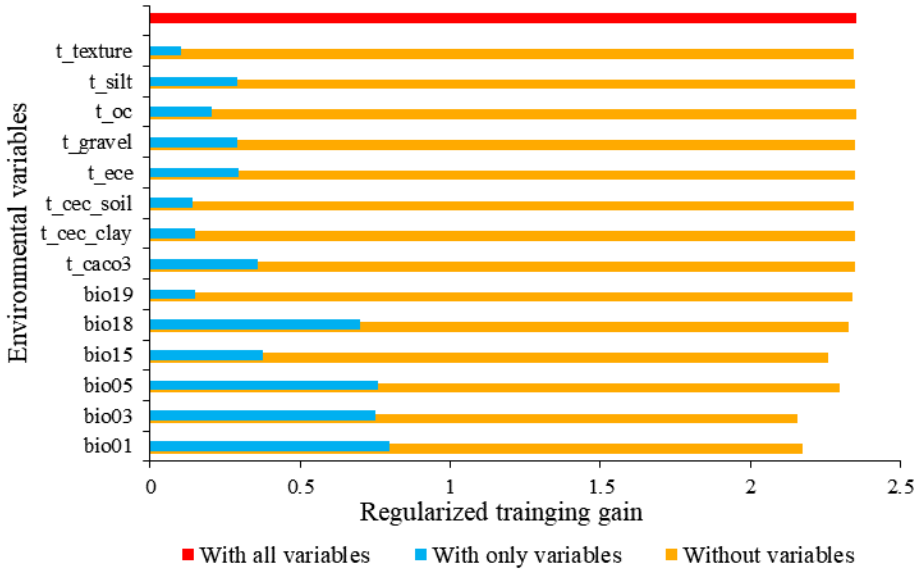

Figure S2. Jackknife of regularized training gain for chili pepper.

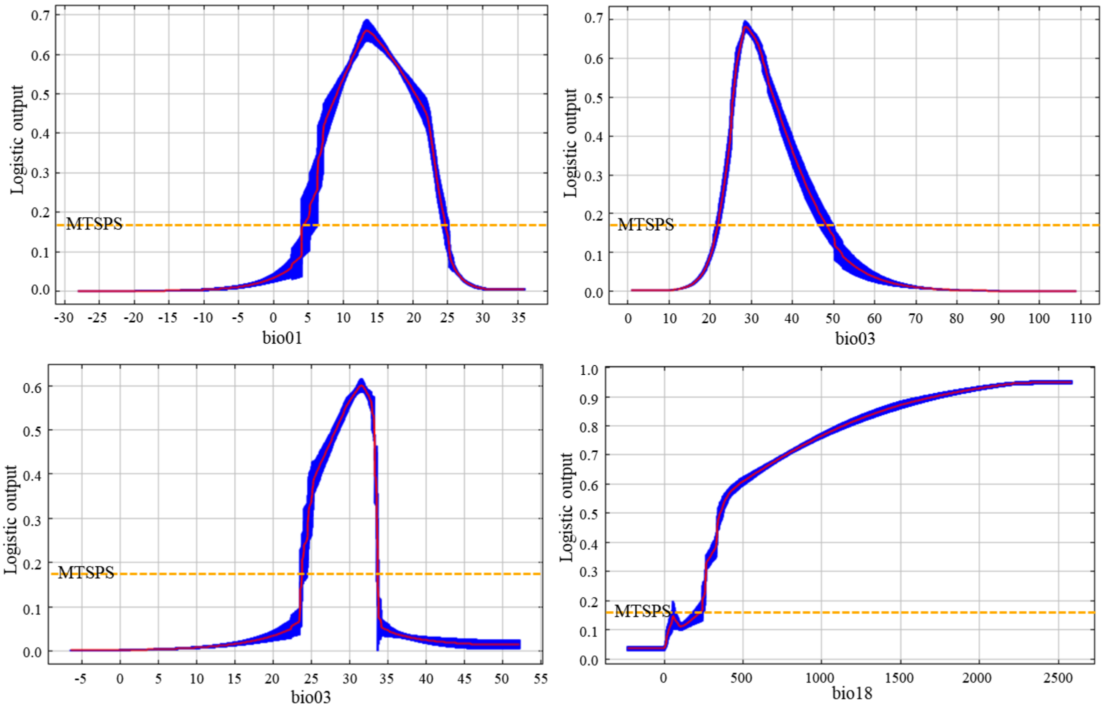

Figure S3. Response curves of the critical environmental variables.
